# Atomic force microscopy identifies the alteration of rheological properties of the cardiac fibroblasts in idiopathic restrictive cardiomyopathy

**DOI:** 10.1371/journal.pone.0275296

**Published:** 2022-09-29

**Authors:** Mizuki Matsumoto, Hirofumi Tsuru, Hidehiro Suginobe, Jun Narita, Ryo Ishii, Masaki Hirose, Kazuhisa Hashimoto, Renjie Wang, Chika Yoshihara, Atsuko Ueyama, Ryosuke Tanaka, Keiichi Ozono, Takaharu Okajima, Hidekazu Ishida

**Affiliations:** 1 Graduate School of Information Science and Technology, Hokkaido University, Sapporo, Japan; 2 Department of Pediatrics, Osaka University Graduate School of Medicine, Osaka, Japan; 3 Department of Pediatrics, Niigata University School of Medicine, Niigata, Japan; Tokyo Ika Shika Daigaku, JAPAN

## Abstract

Restrictive cardiomyopathy (RCM) is a rare disease characterized by increased ventricular stiffness and preserved ventricular contraction. Various sarcomere gene variants are known to cause RCM; however, more than a half of patients do not harbor such pathogenic variants. We recently demonstrated that cardiac fibroblasts (CFs) play important roles in inhibiting the diastolic function of cardiomyocytes via humoral factors and direct cell–cell contact regardless of sarcomere gene mutations. However, the mechanical properties of CFs that are crucial for intercellular communication and the cardiomyocyte microenvironment remain less understood. In this study, we evaluated the rheological properties of CFs derived from pediatric patients with RCM and healthy control CFs via atomic force microscopy. Then, we estimated the cellular modulus scale factor related to the cell stiffness, fluidity, and Newtonian viscosity of single cells based on the single power-law rheology model and analyzed the comprehensive gene expression profiles via RNA-sequencing. RCM-derived CFs showed significantly higher stiffness and viscosity and lower fluidity compared to healthy control CFs. Furthermore, RNA-sequencing revealed that the signaling pathways associated with cytoskeleton elements were affected in RCM CFs; specifically, cytoskeletal actin-associated genes (*ACTN1*, *ACTA2*, and *PALLD*) were highly expressed in RCM CFs, whereas several tubulin genes (*TUBB3*, *TUBB*, *TUBA1C*, and *TUBA1B*) were down-regulated. These results implies that the signaling pathways associated with cytoskeletal elements alter the rheological properties of RCM CFs, particularly those related to CF–cardiomyocyte interactions, thereby leading to diastolic cardiac dysfunction in RCM.

## Introduction

Restrictive cardiomyopathy (RCM) is characterized by normal ventricular wall thickness and motion with low ventricular extensibility, leading to diastolic dysfunction. Pediatric RCM has a very poor prognosis and a 2-year transplant free survival rate of ~40% [1, 2]. In addition, cardiomyocytes in RCM have been extensively investigated from both genetic and mechanical points of view [3, 4]. However, pathogenic sarcomere gene variants are not identified in more than a half of patients with RCM, and thus, the pathogenesis of diastolic dysfunction remains unclear [5–7].

The heart is mainly composed of cardiomyocytes and cardiac fibroblasts (CFs), both of which closely interact via direct cell–cell adhesion, extracellular matrix (ECM), and paracrine factors. Previous studies demonstrate that both cardiomyocytes and CFs play an important role in cardiac function maintenance and development [8, 9]. In addition, we have recently demonstrated that CFs derived from patients with RCM could deteriorate diastolic function of healthy cardiomyocytes via paracrine signaling and direct cell–cell contact [10]. Furthermore, we identified some cytokines and chemokines that might be associated with paracrine CF–cardiomyocyte interactions; however, direct cell–cell interactions are less understood in cardiomyopathies. Since cell stiffness affects neighboring cells [11–13], cellular mechanics in pathological situations needs to be elucidated. Atomic force microscopy (AFM) is a useful tool for evaluating the rheological properties of single cells [14]. In this study, using a frequency-domain AFM rheological method [14–16], we investigated the rheological properties of RCM-derived CFs and compared them with those of healthy CFs.

## Materials and methods

### Harvest and culture of CFs

Three independent primary culture lines of patient-derived CFs (RCM 1, RCM 2, and RCM 3) were obtained at ventricular assist device implantation or heart transplantation, as previously described [10]. Then, the left ventricular specimens were minced and cultured in a cell culture dish with Dulbecco’s modified Eagle’s medium (DMEM) supplemented with 10% fetal bovine serum (FBS) and 1% penicillin/streptomycin. We confirmed the purity of CFs via immunostaining for vimentin, cardiac troponin T, von Willbrand factor, and smooth muscle myosin heavy chains. Healthy CFs (13, 25, and 30 years) were purchased from PromoCell (Heidelberg, Germany) and maintained in the same manner as RCM CFs. Passage number 4 to 7 of the cells were used for all experiments.

### Whole exome sequencing

Genomic DNA was extracted from patients’ peripheral blood samples, and custom-targeted gene enrichment and DNA library preparation were performed using a Nextera Capture Custom Enrichment kit (Illumina, San Diego, CA, USA). DNA samples were analyzed via targeted next-generation sequencing of 257 genes related to cardiomyopathies, and sequenced using the Illumina MiSeq platform, generating approximately two million 150-bp paired-end reads for each sample (Q30 ≥ 90%), as previously described [10]. Genetic variants predicted to alter proteins were selected considering the phenotype prevalence in the general population. To assess the potential functional impacts of variants, we used HGMD, Intervar, CADD, and Protein Variation Effect Analyzer (Provean). We assessed their pathogenic roles by referring to published data and/or evidence from the ClinVar and HGMD databases.

### AFM measurement

We used a customized AFM attached to an upright optical microscope (Eclipse FN1; Nikon, Tokyo, Japan) similar to that reported previously [11, 17, 18]. The deflection of a rectangular silicon nitride cantilever with a sharp silicon tip (BioLever mini, BL-AC40TSC2; Olympus, Tokyo, Japan) [19] was detected by a position-sensitive detector (PSD) measuring the position of the laser beam via a water-immersed objective lens (CFI Plan Fluor 10xW, Nikon). The loading force was determined using Hooke’s law by multiplying the cantilever deflection with the spring constant, which was calibrated using a thermal fluctuation method. The spring constant of the cantilever was less than 0.1 N/m.

**Fig 1:**
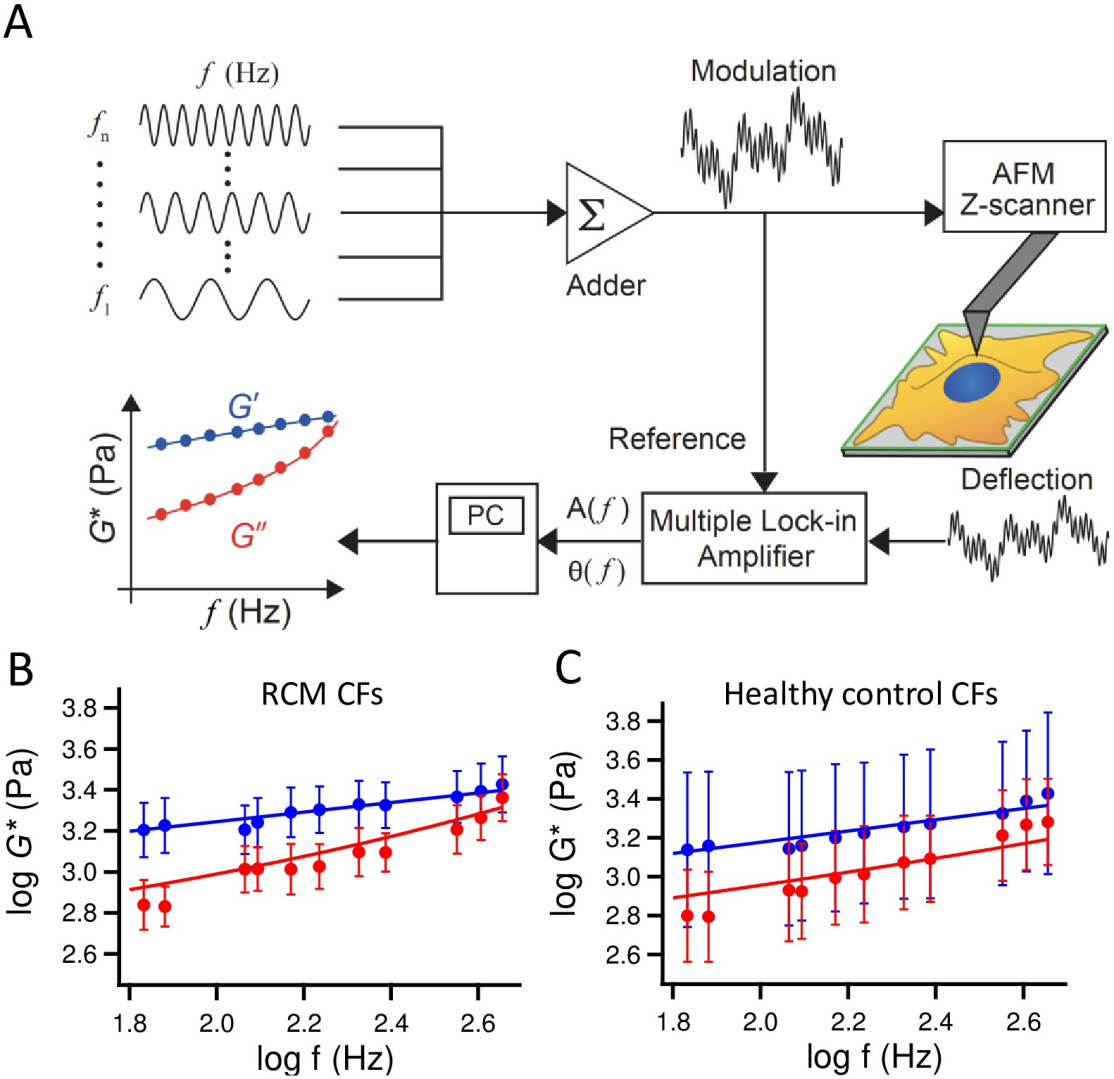
Frequency-domain AFM measurement of cardiac fibroblasts (CFs). (A) Modulated voltages with *n* frequencies from *f* = *f*_1_ to *f*_n_ were added and applied to the z-scanner to oscillate the cantilever. The amplitude *A*(*f*) and phase shift *θ*(*f*) of the cantilever deflection signal at each frequency during the indentation were calculated with a multiple lock-in amplifier program and used to estimate the storage *G’* and loss *G”* moduli as a function of *f* at each measurement position. The frequency dependences of geometric mean and standard deviation of *G’* and *G”* of all cells derived from three patients with RCM (B, n = 165 cells) and three healthy controls (C, n = 63 cells). Solid lines represent the fitted results to Eq 3.

In the frequency-domain AFM rheological measurement, the sample stage approached the cantilever at a constant speed until it reached the maximum loading force of 500 pN, and the cantilever was vibrated with a sinusoidal modulation signal with multiple frequencies, such as *f* = 61, 83, 177, 203, 263, 427, 563, and 611 Hz, and an amplitude of 6 nm using a piezoactuator. The amplitude and phase shift of cantilever displacement with respect to the reference modulation signal at each frequency were acquired using a multiple lock-in amplifier based on a LabVIEW-FPGA program (National Instruments, Austin, TX, USA; Fig 1A). The AFM measurements were performed around the center of the cells, which is conventional in AFM indentation experiments [20]. It is noted that the center location of each cell adhered to a substrate cannot be precisely determined since the cell shapes are not homogeneous. Furthermore, cells exhibit large spatial variations in their rheological properties [21]. Thus, we estimated the rheological properties of the cells obtained from regions around the cell center [20]. Based on the Sneddon’s model for the conical indenter [22], we estimated the diameter of the contact area as less than 2 μm and thus defined the scan area of 10 μm × 10 μm (5 pixels × 5 pixels) around the cell center. The obtained data were averaged to estimate the cell rheological properties. Since our AFM system did not implement a fully shielded incubation system, we used CO_2_-independent medium (Thermo Fisher Scientific, Waltham, MA, USA) and set the temperature at 30°C to reduce the evaporation of the culture medium and minimize the change in the chemical composition of the medium. We confirmed that the mean value of the complex shear modulus G*of cells exhibited no remarkable change over the duration of AFM experiments (~1 h).

In order to estimate G*, we used the Sneddon’s model [22], approximately expressed as follows:

$$F^{*}=\frac{2\text{tan}\theta }{\pi (1-v^{2})}(E_{0}\delta_{0}{}^{2}+2E_{1}{}^{*}\delta_{0}\delta_{1}{}^{*}),$$ (1)

where F* (which is a complex function, as indicated by the asterisk) is the loading force with a small amplitude indentation oscillation, *δ*_1_*, around an operating indentation, *δ*_0_, and *E*_0_ is the Young’s modulus at zero frequency obtained from the approach force curve [16, 17, 21, 23–25]. *θ* is the half-opening angle of the conical indenter, which was set to 17.5° [19]. We assumed the Poisson’s ratio of cell *v* to be 0.5. The frequency-dependent Young’s modulus *E*_1_* is given by 2(1+*ν*)G*. Eliminating the hydrodynamic drag force *F*_d_* given by *F*_d_*/*δ*_1_* = *ib*(0)*f*, where *i* is the imaginary unit, and *b*(*h*) is a viscous drag factor that depends on the separation distance *h* between the cell surface and the probe [26], we obtain G* of the cells as follows:

$$G^{*}=G\prime +iG\prime \prime =\frac{\pi (1-\text{v})}{8\delta_{0}\text{tan}\theta }[\frac{F_{1}{}^{*}}{\delta_{1}{}^{*}}-ib(0)f],$$ (2)

where *G’* and *G”* represent the storage and loss moduli of the cell, respectively, and *F*_1_* = 4tan*θ* · *E*_1_*δ_0_*δ*_1_*/{π(1 − *v*^2^)}. The *b*(0) value was determined by extrapolating the *b* (*h*) values measured at various separation distances at *f* = 100 Hz.

AFM data were analyzed using Igor Pro software (WaveMetrics, Portland, OR, USA). For each cell, *G’* and *G”* as a function of *f* were fitted to the power-law structural damping model with additional Newtonian viscosity [16, 17, 23–25, 27–29] given by

$$G^{*}=G_{0}g(\alpha )\{1+i\eta (\alpha )\}{\left(\frac{f}{f_{0}}\right)}^{\alpha }+i\mu f,$$ (3)

where *α* is the power-law exponent showing between 0 for solid and 1 for liquid. *g*(*α*) is *Γ*(1 − *α*) cos(πα/2), with *Γ* denoting the gamma function. *G*_0_ is the scale factor of the modulus at a frequency scale factor *f*_0_, which was arbitrarily set to 1 Hz. The hysteresivity η(*α*) is equivalent to tan(π*α*/2) and *μ* is the Newtonian viscous damping coefficient. It is reasonable to assume that the Newtonian viscous damping coefficient of living cells should not be a negative value, thus in our analysis, we defined a constraint that *μ* is kept at not having a negative value.

### RNA-sequencing and gene expression analysis

Total RNA was extracted from each CF line and sequenced using the Illumina HiSeq 2500 platform, as previously described [10]. Then, sequenced reads were mapped to the human reference genome sequence (hg 19) using TopHat ver. 2.0.13 in combination with Bowtie2 ver. 2.2.3 and SAMtools ver. 0.1.19. Subsequently, fragments per kilobase of exon per million mapped fragments (FPKMs) were calculated using Cuffnorm ver. 2.2.1. Integrated differential expression and pathway analysis (iDEP) software was used for gene expression analyses. Gene expression levels based on calculated FPKMs were statistically compared between three lines of RCM CFs and three lines of healthy CFs using unpaired two-tailed *t*-test.

### Immunocytochemical analyses of CFs

Cells were fixed using 4% paraformaldehyde/phosphate-buffered saline (PBS), and were then permeabilized by 0.1% Triton-X/PBS for 10 min. For staining actin filaments and microtubules, cells were incubated overnight at 4°C with Alexa Fluor 488-conjugated phalloidin (1:200; A12379, Thermo Fisher Scientific) and anti-tubulin primary antibody (1:500; 14-4502-82, Thermo Fisher Scientific), followed by blocking with 1% bovine serum albumin. Alexa Fluor 532 secondary antibody (1:500; Thermo Fisher Scientific) was applied at room temperature for 30 min, then nuclei were stained with Hoechst33342 (1:1000; H342, Dojindo Molecular Technologies, Kumamoto, Japan). The stained cell samples were imaged using a laser scanning confocal microscope (C1, Nikon, Tokyo, Japan).

### Ethical statement

This study was approved by the Research Ethics Committee of Osaka University (nos. 15211 and 442). Written informed consent was obtained from the parents of the minors included in this study before obtaining heart specimens and blood samples.

### Statistical analysis

The statistical analyses were performed using JMP Pro 14 software. Unpaired two-tailed *t*-test was performed to compare three individuals of RCM group and three individuals of healthy control. Upon confirming normal distribution by Shapiro-Wilk test, unpaired two-tailed *t*-test was performed to compare AFM data from all cells between RCM and healthy control groups. Otherwise, Mann-Whitney U test was performed. Statistical significance was set at *P* < 0.05.

## Results

### Patients’ profiles

The summary of patients’ clinical profiles is presented in Table 1. Patient’s ages during sampling were 3, 11, and 2 years; all of them underwent ventricular assist device implantation and heart transplantation. All the samples were harvested from the left ventricle: RCM 1 and RCM 2 were identified as *TNNI3* missense variants, which were reported to cause RCM, and no pathological variants were observed in RCM 3. Left ventricle end diastolic pressure (LVEDP) was elevated in all RCM patients. Furthermore, histological analysis of cardiac fibrosis revealed no significant difference in the fibrotic area, as previously described [10].

**Table 1:** Clinical characteristics of patients.

	RCM 1	RCM 2	RCM 3
**Sex**	Male	Male	Female
**Age at diagnosis**	2 years	6 years	8 months
**Age at sampling**	3 years	11 years	2 years
**Event at sampling**	LVAD	LVAD	HTx
**LVEDP**	24 mmHg	24 mmHg	25 mmHg
**Medications at sampling**	Dobutamine	Milrinone	LVAD
	Milrinone	Diuretics	Dobutamine
	Diuretics	ACE inhibitor	Milrinone
	Beta-blocker	Amiodarone	Diuretics
	Aspirin	Aspirin	Warfarin
**BNP at sampling**	568.8 pg/mL	2577.5 pg/mL	949.7 pg/mL
**Pathogenic Gene variants**	*TNNI3* (R170W)	*TNNI3* (R192H)	Not detected

LVEDP, left ventricular end diastolic pressure; BNP, brain natriuretic peptide; LVAD, left ventricular assist device; HTx, heart transplantation; ACE, angiotensin-converting enzyme.

### AFM measurement of rheological properties in RCM-derived CFs

Fig 1B and 1C show the geometric mean and the standard deviation of *G’* and *G”* of all CFs derived from three patients with RCM and three healthy controls, respectively, as a function of frequency, measured by AFM. The standard deviation represents the cell-to-cell variation of G* at frequencies in the same group. The frequency dependence of G* in both RCM and healthy CFs followed the single power-law rheology (PLR) model described in Eq 3, indicating that CFs from three individuals in the same group exhibited similar rheological behaviors. On the other hand, the shape of the curves were different between RCM (Fig 1B) and healthy (Fig 1C) CFs, indicating the existence of differences in rheological parameters between RCM and healthy CFs. In our AFM measurements of RCM and healthy CFs, the indentation depth at the trigger force of 500 pN was typically 1‒2 μm, depending on the stiffness of the cells.

For each cell measured by AFM, we quantified the power-law rheological parameters such as *G*_0_, *α*, and *μ*, which are the cell modulus scale factor, power-law exponent, and Newtonian viscous damping coefficient, respectively. Studies have reported that the number (ensemble) distribution of complex modulus G* in single cells exhibits a log-normal distribution [27, 28, 30, 31], and *G*_0_ and *α* of cells show log-normal and normal (Gaussian) distributions, respectively [21, 24, 25, 32–35]. Thus, in this study, we estimated *G*_0_ in a logarithmic scale and *α* in a linear scale. We confirmed that Shapiro-Wilk test showed that log*G*_0_ and *α* of all cells measured by AFM were normally distributed whereas *μ* was not normal.

**Fig 2:**
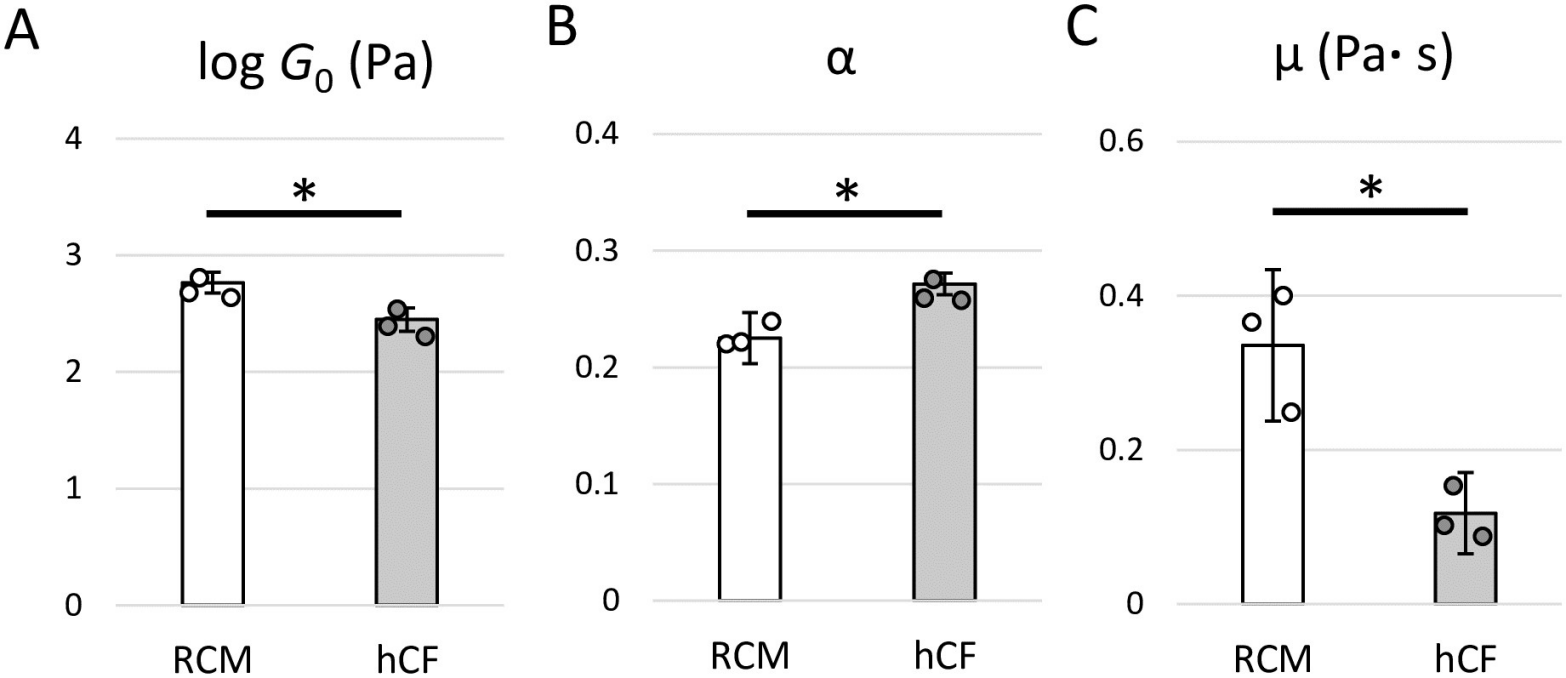
Quantification of power-law rheological parameters of the cardiac fibroblasts (CFs). (A) The cell modulus scale factor, *G*_0_, (B) the power-law exponent, *α*, and (C) the Newtonian viscous damping coefficient, *μ*, in CFs derived from patients with restrictive cardiomyopathy (RCM, n = 3) and healthy controls (hCF, n = 3). Data are presented as mean ± standard deviation. *P < 0.05 by unpaired two-tailed *t*-test.

We performed statistical analyses on rheological properties such as *G*_0_, *α*, and *μ* between three individuals in RCM and healthy CFs with unpaired *t*-test. As a result, the *G*_0_, which is related to cell stiffness [21], was significantly higher in RCM CFs than that in healthy CFs (*P* = 0.0153; Fig 2A), indicating that intracellular structures such as the cytoskeleton of RCM CFs were stiffer compared to those of healthy CFs [11, 21, 25, 27–29]. In addition, we found that *α* was significantly decreased in RCM CFs compared to those in healthy CFs (*P* = 0.0237; Fig 2B). According to the soft glassy rheology [27–29], *α* corresponds to the probability that a system evolves in a complex energy landscape with a high number of traps. Thus, the results shown in Fig 2B suggest that the intracellular structures in RCM were more stable than those in healthy CFs. In contrast, *μ* was significantly elevated in RCM CFs (*P* = 0.0276; Fig 2C), indicating that cytoplasmic components in RCM CFs had a significantly higher Newtonian viscosity than those in healthy CFs.

All of the data acquired from AFM are provided in S1 Fig. Using unpaired *t*-test for log *G*_*0*_ and α, and Mann-Whitney U test for *μ*, we again found that *G*_0_ and *μ* were significantly higher in RCM CFs (*P*<0.001) while *α* was significantly lower in RCM CFs (*P*<0.001), as compared to those in healthy CFs.

### Comprehensive gene expression analyses by RNA-sequencing

**Fig 3:**
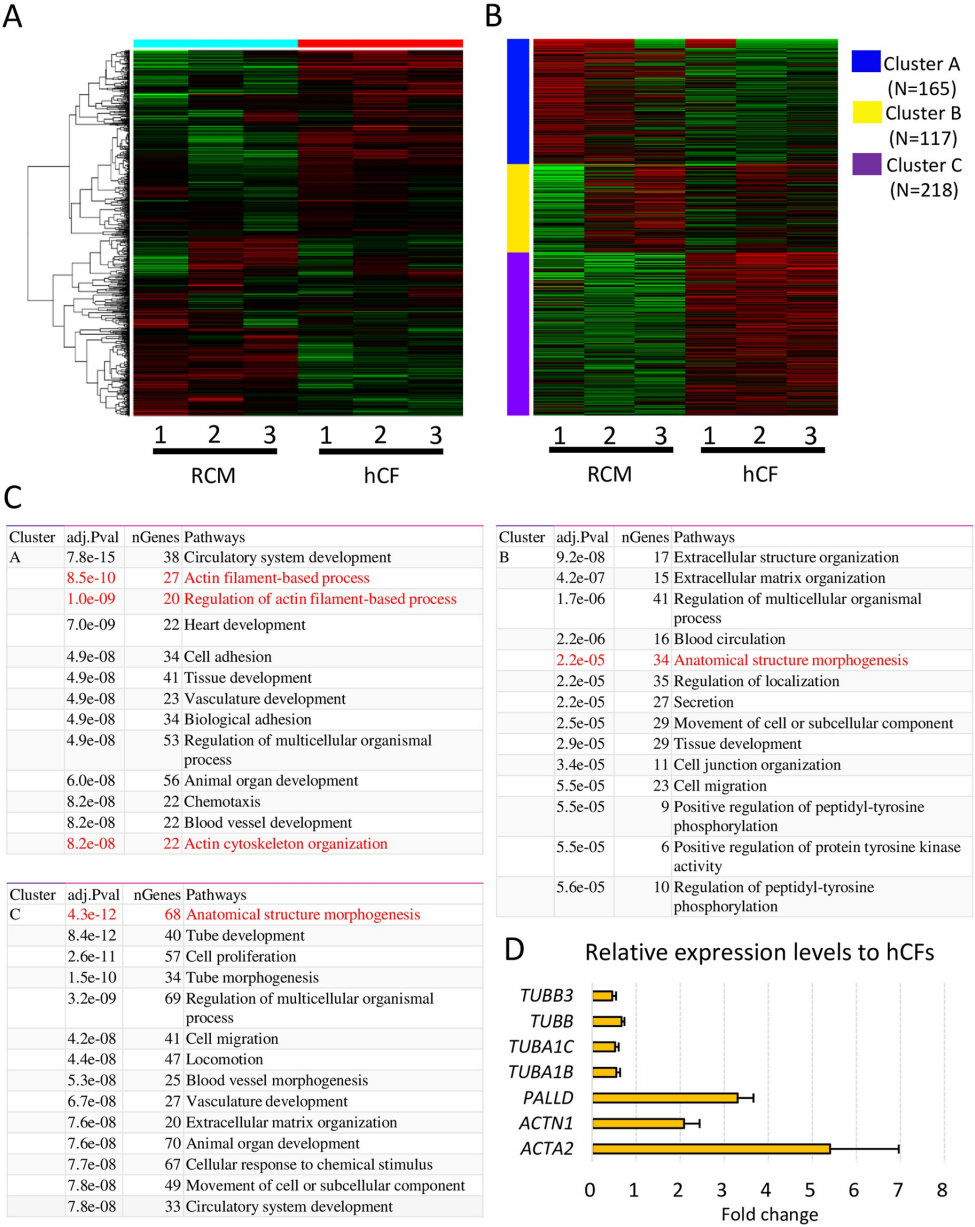
RNA-sequencing analyses of cardiac fibroblasts (CFs). (A) Hierarchical clustering of top 1000 genes between restrictive cardiomyopathy (RCM) and healthy control CFs (hCF). (B) K-means clustering of RCM CFs and hCFs. (C) Pathway analyses of K-means clustering indicate that actin filament-associated processes and anatomical structure morphogenesis are affected in RCM CFs. (D) Differential gene expression analysis revealed that expression levels of several genes associated with the cytoskeleton and cell adhesion are significantly up- or down-regulated in RCM CFs.

To investigate the gene expression profiles associated with cytoskeletal components, we conducted RNA-sequencing using a next-generation sequencer. Heat map analysis of the top 1000 genes showed different expression patterns between RCM and healthy CFs (Fig 3A). K-means clustering also showed difference in expression patterns between RCM and healthy CFs (Fig 3B). Pathway analyses showed that the actin filament-based process, actin cytoskeleton organization, and anatomical structure morphogenesis were significantly altered in RCM CFs compared to healthy CFs (Fig 3C). Differential gene expression analysis also revealed significant differences in the expression of genes associated with anatomical structure morphogenesis and cellular component morphogenesis between the two groups. Notably, the expression of cytoskeletal actin-associated genes (*ACTN1*, *ACTA2*, and *PALLD*) were significantly upregulated in RCM CFs, whereas that of several tubulin genes (*TUBB3*, *TUBB*, *TUBA1C*, and *TUBA1B*) were significantly down-regulated (Fig 3D). These results suggest that alterations in the expression of multiple genes in cytoskeleton components might change the rheological properties of RCM CFs.

### Immunocytochemistry of RCM and healthy CFs

**Fig 4:**
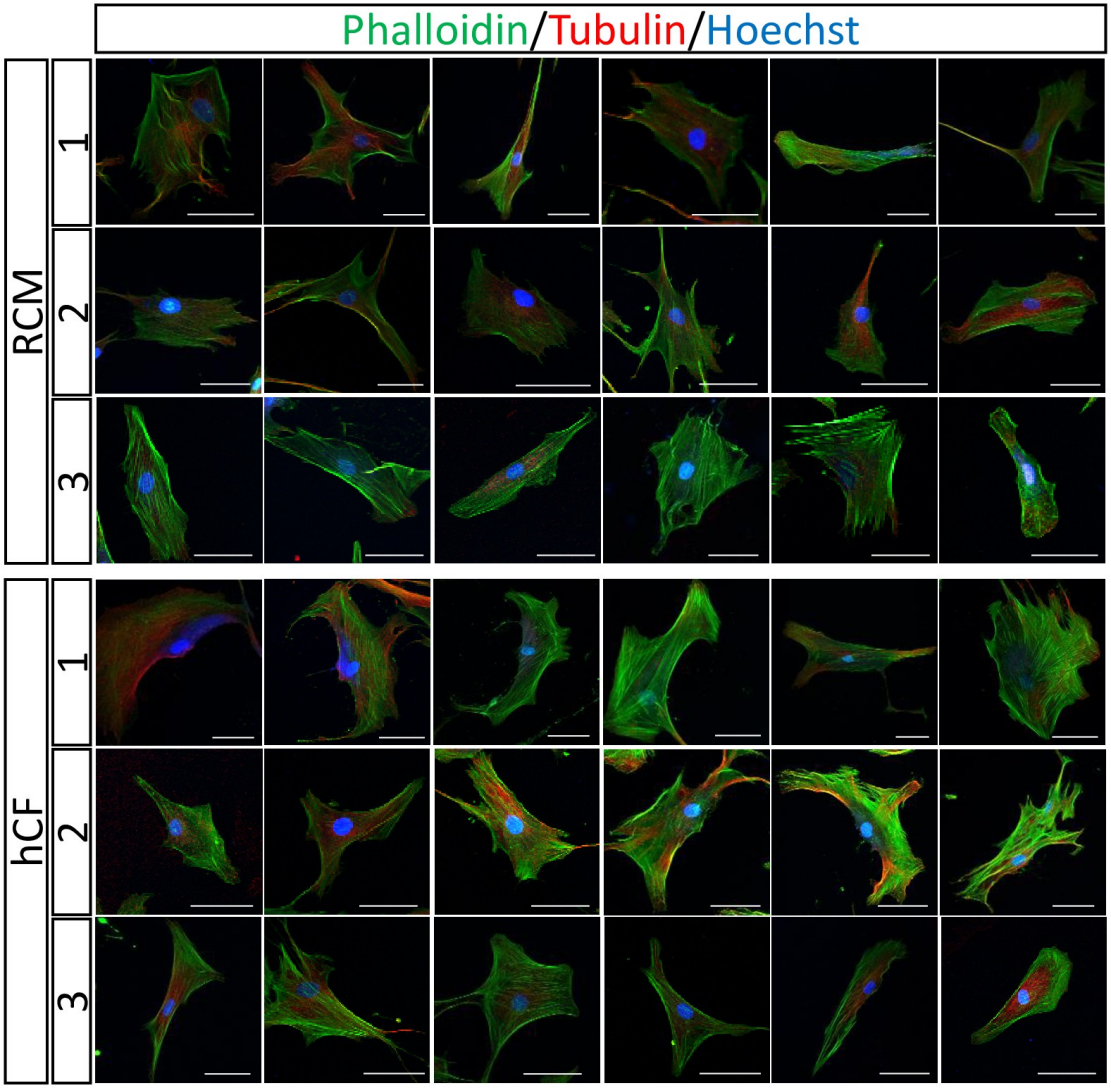
Immunocytochemistry of cellular cytoskeletal components in cardiac fibroblasts (CFs). Representative images of immunocytochemistry for actin filaments (phalloidin) and tubulin in RCM CFs and healthy CFs. The numbers represent the individuals. Scale bar: 50 μm.

To visualize the cellular cytoskeletal components in CFs, we stained actin filaments and microtubules in RCM and healthy CFs (Fig 4). Although the cell shape and the cytoskeletal structures varied among individual cells in both groups, we did not observe any remarkable differences between RCM and healthy CFs in the stained images, indicating that AFM and RNA-sequencing detected subtle alteration of the multiple cytoskeletal components in RCM CFs that could not be delineated through fluorescent observations.

## Discussion

RCM is a unique type of cardiomyopathy that presents with preserved contractile function and severe disturbance in diastolic function. Previous studies using the overexpression models of *TNNI3* mutants indicated that the molecular pathogenesis of diastolic dysfunction in RCM might be caused by Ca^2+^ hypersensitivity in cardiomyocytes [36, 37]. However, more than a half of RCM patients could not be identified as candidate variants in sarcomere genes despite dedicated analysis via whole exome sequencing [27, 38]. Moreover, no significant differences were found in clinical manifestations between patients with or without sarcomere mutations [10]. Thus, these RCM clinical features suggest that some non-cardiomyocyte-derived factors may be associated with RCM pathogenesis. The heart consists of not only cardiomyocytes but also other supportive cells, mainly CFs. Several studies have demonstrated that CFs play important roles in cardiac development and healing after injury [8, 9]. CFs communicate with each other and maintain healthy cardiac function [39]. We also reported that RCM CFs could deteriorate the diastolic function of healthy cardiomyocytes via both humoral factors and direct cell–cell contact [10].

In this study, we demonstrated using AFM, that the rheological properties of RCM CFs were significantly altered compared to those of healthy CFs. Since CFs act as scaffolds for cardiomyocytes in the heart and account for approximately 60% of the cells in the heart [8, 9], we speculate that the mechanical properties of CFs affect the functioning of cardiomyocytes and the mechanical properties of the whole heart. Cellular mechanistic changes affect the behavior of neighboring cells [11–13] and the microenvironment of the cellular network [40–42]. In fact, a previous study on dilated cardiomyopathy-derived CFs demonstrated that the apparent Young’s modulus measured by AFM correlated with left ventricular dilatation [43]. Another recent study demonstrated that increased cardiac stiffness evaluated by AFM is correlated with the functional decline of the heart in a zebrafish model [44]. Based on the results obtained in our study, we propose a scenario wherein the mechanical properties of CFs affect the behavior of cardiomyocytes, thereby suggesting its association with the pathogenesis of RCM.

In histological analysis, cardiac fibrosis has commonly occurred in RCM patients [10]. In the process of deteriorating diastolic function of the heart, the cytoskeleton and ECM exhibit highly fibrotic structures. It has been recognized that cells attached to ECM can sense the stiffness of substrates and thereby increase the cell stiffness [45, 46] as well as the cell tension [47, 48] by actomyosin activation, often affecting gene expression [49, 50]. The increase of cell stiffness or cell tension by activating actomyosin decreases cell fluidity, which is correlated with the increase in the prestress and external stresses [29]. In RCM patients, ventricular end diastolic pressure is elevated compared to that in the healthy heart (Table 1) [51]. Taken collectively from our AFM results showing that RCM CFs exhibited increased stiffness and decreased fluidity, we speculate that the mechanical properties of RCM CFs are regulated by the *in vivo* pathological environment including hemodynamic stress around the CFs as well as the neighboring cardiomyocytes, thereby suggesting that the mechanical feedback among cardiomyocytes, ECM, and CFs can be crucial to elucidate the pathological mechanisms of RCM. The hemodynamic mechanical stress may affect the gene expression profiles of cytoskeletal components in CFs.

In addition to AFM mechanical assessments, we conducted RNA-sequencing to reveal the comprehensive gene expression profiles of RCM CFs and healthy CFs. Interestingly, RCM CFs showed highly different expression patterns compared to healthy CFs. Furthermore, K-means clustering revealed that several signaling pathways associated with the cellular structure and cytoskeleton were altered in RCM CFs relative to healthy CFs. We identified several specific gene expressions associated with the cytoskeleton in RCM CFs. Actinin alpha 1, actin alpha 2, and palladin are the major components of the actin cytoskeleton [52]. The actin alpha 2 expression is upregulated when fibroblasts are activated in pathological situations and cell behavior regulation [53]. Palladin regulates actin-associated microfilaments and is correlated with cardiovascular diseases and cancer [54, 55]. In contrast, several tubulin-associated genes were downregulated in RCM CFs relative to healthy CFs. Tubulins comprise microtubules that are important components of the cytoskeleton [56, 57]. It has been reported that actin disruption can decrease cell stiffness, whereas tubulin disruption can increase cell stiffness [58]. These transcriptome findings regarding actin- and tubulin-associated genes may correspond to rheological analyses via AFM that RCM CFs have higher stiffness compared to healthy CFs. However, we observed no remarkable differences between RCM and healthy CFs in the stained images, suggesting that AFM detected a subtle difference in the formation and remodeling of cytoskeletal structures, not in the cytoskeletal density.

We observed with AFM that RCM CFs exhibited a higher Newtonian viscous damping coefficient that corresponds to the linear viscosity of cell cytoplasm. Previous studies have reported that the Newtonian viscous damping coefficient was less sensitive to the modifications of cytoskeleton structures [27, 28], indicating that the intracellular components except the cytoskeleton may be the main factors in changing the linear viscous component of cell cytoplasm. However, the origin of cell components affecting the linear viscosity has not been fully understood.

Overall, our results indicate that multiple alterations in the cytoskeletal gene expression levels affect the rheological properties of RCM CFs. The most critical limitation of this study is the small sample size of the patients, owing to the rarity in the occurrence of RCM. Therefore, further investigation is warranted with larger sample size to substantiate our findings on AFM data and gene expression profiles of RCM CFs, and to directly clarify how the alterations of gene expressions in RCM CFs could affect the AFM measurements.

## Conclusion

We investigated the gene expression profiles and the mechanical properties of CFs derived from patients with RCM. The RNA-sequencing showed that cytoskeletal actin-associated genes were highly expressed in CFs derived from patients with RCM, whereas several tubulin genes were down-regulated. The frequency-domain AFM showed that CFs derived from patients with RCM had higher stiffness and viscosity and lower fluidity than those derived from healthy CFs, indicating an association with the formation and remodeling of cytoskeletal structures. Our results suggest a possible relationship between the gene expression and the mechanical feature of RCM CFs.

## Supporting information

S1 Fig All data of power-law rheological parameters in the cardiac fibroblasts (CFs). The dots represent all data of (A) the cell modulus scale factor, *G*_0_, (B) the power-law exponent, *α*, and (C) the Newtonian viscous damping coefficient, *μ*, in CFs derived from patients with restrictive cardiomyopathy (n = 165 cells, RCM 1: number of the cells were 88; RCM 2: number of the cells were 30; RCM 3: number of the cells were 47) and healthy controls (n = 63 cells, hCF 1: number of the cells were 20; hCF 2: number of the cells were 16; hCF 3: number of the cells were 27). *P<0.001 by unpaired two-tailed *t*-test and **P<0.001 by Mann-Whitney U test.
(PDF)
