# Supplementary figures and images for: Atomic force microscopy identifies the alteration of rheological properties of the cardiac fibroblasts in idiopathic restrictive cardiomyopathy
Source: PLoS One. 2022 Sep 29;17(9):e0275296. doi: 10.1371/journal.pone.0275296 (PMC9522286; doi:10.1371/journal.pone.0275296)

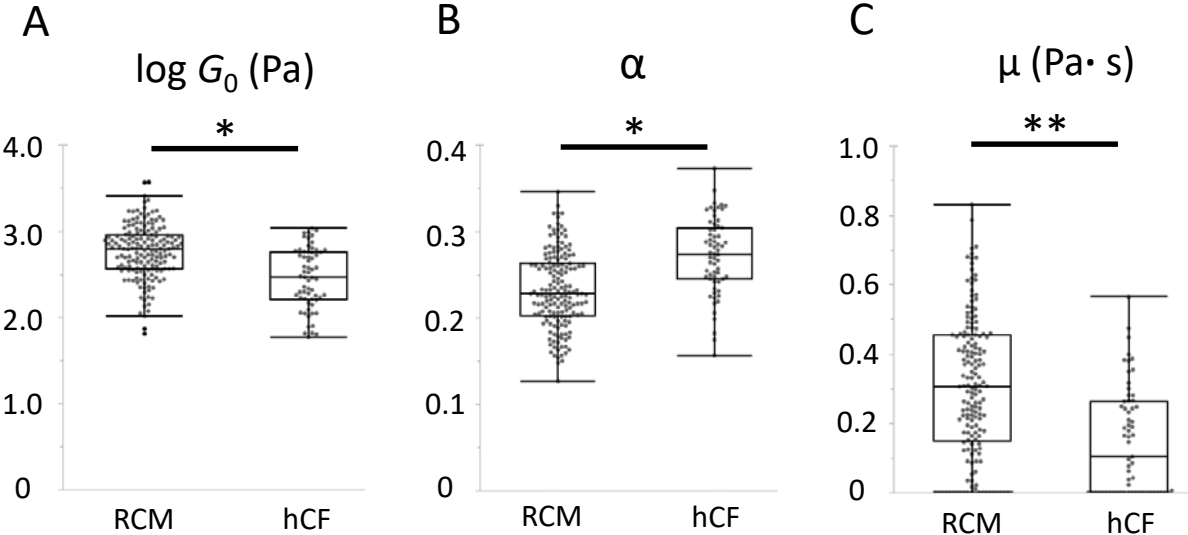

Supplement: S1 Fig — The dots represent all data of (A) the cell modulus scale factor, G0, (B) the power-law exponent, α, and (C) the Newtonian viscous damping coefficient, μ, in CFs derived from patients with restrictive cardiomyopathy (n = 165 cells, RCM 1: number of the cells were 88; RCM 2: number of the cells were 30; RCM 3: number of the cells were 47) and healthy controls (n = 63 cells, hCF 1: number of the cells were 20; hCF 2: number of the cells were 16; hCF 3: number of the cells were 27). *P<0.001 by unpaired two-tailed t-test and **P<0.001 by Mann-Whitney U test. (PDF) [file pone.0275296.s001.pdf]
